# Screening of 109 neuropeptides on ASICs reveals no direct agonists and dynorphin A, YFMRFamide and endomorphin-1 as modulators

**DOI:** 10.1038/s41598-018-36125-5

**Published:** 2018-12-20

**Authors:** Anna Vyvers, Axel Schmidt, Dominik Wiemuth, Stefan Gründer

**Affiliations:** Institute of Physiology, RWTH Aachen University, Pauwelsstrasse 30, D-52074 Aachen, Germany

## Abstract

Acid-sensing ion channels (ASICs) belong to the DEG/ENaC gene family. While ASIC1a, ASIC1b and ASIC3 are activated by extracellular protons, ASIC4 and the closely related bile acid-sensitive ion channel (BASIC or ASIC5) are orphan receptors. Neuropeptides are important modulators of ASICs. Moreover, related DEG/ENaCs are directly activated by neuropeptides, rendering neuropeptides interesting ligands of ASICs. Here, we performed an unbiased screen of 109 short neuropeptides (<20 amino acids) on five homomeric ASICs: ASIC1a, ASIC1b, ASIC3, ASIC4 and BASIC. This screen revealed no direct agonist of any ASIC but three modulators. First, dynorphin A as a modulator of ASIC1a, which increased currents of partially desensitized channels; second, YFMRFamide as a modulator of ASIC1b and ASIC3, which decreased currents of ASIC1b and slowed desensitization of ASIC1b and ASIC3; and, third, endomorphin-1 as a modulator of ASIC3, which also slowed desensitization. With the exception of YFMRFamide, which, however, is not a mammalian neuropeptide, we identified no new modulator of ASICs. In summary, our screen confirmed some known peptide modulators of ASICs but identified no new peptide ligands of ASICs, suggesting that most short peptides acting as ligands of ASICs are already known.

## Introduction

Acid-sensing ion channels form a small family of proton-gated ion channels that belongs to the degenerin/epithelial Na^+^ channel (DEG/ENaC) gene family^1^. There are four genes coding for ASICs, ACCN1-ACCN4, plus a closely related gene coding for an ion channel that is sensitive to bile acids and has therefore been named bile acid-sensitive ion channel (BASIC)^2^; alternatively, BASIC is called ASIC5. ASICs are trimers^3,4^. Homomeric ASIC1a and ASIC3 have a high proton sensitivity (EC_50_ ~pH 6.5); homomeric ASIC1b, a splice variant of ASIC1a, has a lower proton sensitivity (EC_50_ ~pH 5.9) and homomeric ASIC2a has a very low proton sensitivity (EC_50_ < pH 4.5)^5–7^. Homomeric ASIC2b, a splice variant of ASIC2a, is not activated by low pH, but contributes to heteromeric channels^8^. The physiological stimuli of ASIC4 and of ASIC5 are unknown^9,10^.

Some neuropeptides bind to ASICs and modulate their function^11^. For example, FMRFamide and several FMRFamide-related peptides, including the mammalian neuropeptides FF (NPFF; FLFQPQRFamide) and AF (NPAF; AGEGLSSPFWSLAAPQRFamide)^12^ and the conorfamide RPRFamide^13^, probably bind to a cavity in the lower palm domain^14^ and slow desensitization of ASIC3^12,13^. Other examples are some endogenous opioid peptides, like endomorphin-1 (YPWFamide) and endomorphin-2 (YPFFamide), which slow desensitization of ASIC3^15^, and big dynorphin, which limits steady-state desensitization of ASIC1a^16^.

Interestingly, other channels in the DEG/ENaC gene family are directly activated by neuropeptides. The first peptide-gated channels to be characterized, the FMRFamide-activated Na^+^ channel (FaNaC) from snails, and the Hydra Na^+^ channels (HyNaCs) from Hydra, are activated by RFamide neuropeptides^17–19^. The recent discovery of a DEG/ENaC activated by a Wamide, the myoinhibitory peptide gated ion channel (MGIC), which is closely related to FaNaC, however, demonstrated that peptides activating DEG/ENaCs are heterogenous^20^. Modulation of ASICs by neuropeptides and direct activation of peptide-gated DEG/ENaCs by neuropeptides render peptides candidate agonists also for ASIC4 and BASIC.

In this study, we performed an unbiased screen of 109 neuropeptides that contain <20 amino acids. We assessed, first, whether any of the 109 peptides could directly activate ASICs, in particular ASIC4 and BASIC, and, second, whether any of the 109 peptides modulated gating of homomeric ASIC1a, homomeric ASIC1b or homomeric ASIC3.

## Results

We selected 109 neuropeptides from the online database NeuroPep (http://isyslab.info/NeuroPep/)^21^ that met the following criteria: first, organism: *Rattus*, second, sequence length: below 20 amino acids. To search for potential modulatory effects of neuropeptides on ASIC1a, ASIC1b and ASIC3, we pooled the 109 neuropeptides in 13 mixes as indicated in Table 1. We designed an experimental protocol which captures effects of neuropeptides on both activation and steady-state-desensitization: ASICs were preconditioned with a pH that desensitizes 50% of the channels and were then activated with a pH that opens 50% of the remaining channels, which were not desensitized. Thus, 50% of the ASICs were desensitized, 25% were opened and 25% stayed closed during activation by low pH. We alternately applied preconditioning solutions with or without peptides and looked for changes in current amplitude and desensitization kinetics in the presence of the peptides. Pre-application of peptides would also reveal a direct activation of the channels.

**Table 1:** Composition of the 13 peptide mixes screened in this study.

Name	Amino acid sequence	Name	Amino acid sequence
Mix 1		Mix 7	
Macrophage inhibitory factor	TKP	7B2 (SCG5; Secretogranin-V) C-terminal peptide	SVPHFSEEEKEPE
Thyrotropin-releasing hormone precursor	QHP-NH_2_	Neurotensin	pQLYENKPRRPYIL
Thyrotropin-releasing hormone	pQHP-NH_2_	Apelin-13	QRPRLSHKGPMPF
N-Tyr-Melanocyte-inhibiting factor (MIF-1)	YPLG-NH_2_	Rimorphin	YGGFLRRQFKVVT
Angiotensin I/II(1–4)	DRVY	DynorphinA(1–13)	YGGFLRRIRPKLK
Endomorphin-1	YPWF-NH_2_	α-Melanocyte-stimulating hormone	SYSMEHFRWGKPV-NH_2_
Leu-enkephalin	YGGFL	Pro-Melanin-concentrating hormone(131–144)	EIGDEENSAKFPIG
Met-enkephalin	YGGFM	Propeptide21–45(1–14)	QPVVPVEAVDPMEQ
long proCART(55–59)	IPIYE	Secretogranin I(186–199)	HTEESGEKHNAFSN
Angiotensin I/II(1–5)	DRVYI	Secretogranin II(599–612)	LNQEQAEQGREHLA
**Mix 2**		**Mix 8**	
Tyr-FMRFamide	YFMRF-NH_2_	WE-14	WSRMDQLAKELTAE
Neuromedin-N	KIPYIL	Urotensin 2	QHGTAPECFWKYCI
NT-2 (fragment analogue of neurotensin)	RKPWLL	Somatostatin 14	AGCKNFFWKTFTSC
Angiotensin IV	VYIHPF	Secretogranin I(437–451)	DEGHDPVHESPVDTA
ORG2766	(M-O)EHF(D-K)F	VGF(180–194)	pQQETAAAETETRTHT
Selank	TKPRPGP	Thymosin beta-10(30–44)	PTKETIEQEKRSEIS
KEP	ARPVKEP	Secretogranin II(598–612)	YLNQEQAEQGREHLA
ACTH(4–10)	MEHFRWG	[des-Ser1]-cerebellin	GSAKVAFSAIRSTNH
Angiotensin I/II(1–7)	DRVYIHP	BigLEN	LENSSPQAPARRLLPP
Propeptide 219–229(1–7)	VGRPEWW	VGF(220–235)	VPERAPLPPSVPSQFQ
**Mix 3**		**Mix 9**	
Angiotensin III	RVYIHPF	Secretogranin III(38–53)	ELSAERPLNEQIAEAE
Met-enkephalin-RGL	YGGFMRGL	Cerebellin	SGSAKVAFSAIRSTNH
Substance P-like	RPQQFGLM -NH_2_	Neuropeptide AF-like	AGEGLSSPFWSLAAPQR-NH_2_
Dynorphin A(1–8)	YGGFLRRI	Thymosin beta-4(28–44)	PLPSKETIEQEKQAGES
Neuropeptide FF	FLFQPQRF-NH_2_	Chromogranin A(377–393)	LEGEDDPDRSMKLSFRA
Angiotensin II	DRVYIHPF	Neuropeptide2 (NocII)	FSEFMRQYLVLSMQSSQ
Oxytocin	CYIQNCPLG-NH_2_	Nociceptin	FGGFTGARKSARKLANQ
7B2 (SCG5; Secretogranin-V) C-terminal peptide(5–13)	FSEEEKEPE	Dynorphin A(1–17)	YGGFLRRIRPKLKWDNQ
Secretogranin I(454–462)	YPQSKWQEQ	LittleSAAS	SLSAASAPLAETSTPLRL
Bradykinin	RPPGFSPFR	Tubulin beta-4B chain(168–185)	SVVPSPKVSDTVVEPYNA
**Mix 4**		**Mix 10**	
Gonadotropin-releasing hormone-1-like	HWSYGLRPG	Octadecaneuropeptide	QATVGDVNTDRPGLLDL(K-Ac)
β-neoendorphin	YGGFLRKYP	Neuropeptide NPVF	ANMEAGTMSHFPSLPQRF-NH_2_
Arg-vasopressin	CYFQNCPRG-NH_2_	β-Melanocyte-stimulating hormone	ADGPYRVEHFRWGNPPKD
Angiotensin I(1–9)	DRVYIHPFH	Neuroendocrine regulatory peptide-4	NAPPEPVPPPRAAPAPTHV
LittleLEN	LENSSPQAPA	Neuropeptide-GE	GPAVFPAENGVQNTESTQE
Secretogranin I(371–380)	SEESQEKEYP	long proCART(10–28)	ALDIYSAVDDASHEKELPR
HFHH-10	HFHHALPPAR	LQEQ-19	LQEQEELENYIEHVLLHRP
Antrin	APSDPRLRQF		
Gonadotropin-releasing hormone-1	pQHWSYGLRPG-NH_2_		
Substance P-like	RPKPQQFGLM-NH_2_		
**Mix 5**		**Mix 11**	
α-neoendorphin	YGGFLRKYPK	VGF(211–217)	ASWGEFQ
Kisspeptin-10	YNWNSFGLRY-NH_2_	Cholecystokinin-8	DYMGWMDF-NH_2_
[Ser2]-Neuromedin-C	GSHWAVGHLM-NH_2_	Neurokinin-B	DMHDFFVGLM-NH_2_
Secretogranin-I (585–594)	SFAKAPHLDL	Proenkephalin 114–133(7–20)	VEPEEEANGGEILA
Neurokinin-A	HKTDSFVGLM-NH_2_	Chromogranin A(377–391)	LEGEDDPDRSMKLSF
Angiotensin I	DRVYIHPFHL	β-Preprotachykinin	ALNSVAYERSAMQNYE
Chromogranin A derivative	AYGFRDPGPQL	Gastrin	pQRPPMEEEEEAYGWMDF
TLQP-11 (VGF-derived peptide)	TLQPPASSRRR		
Neuropeptide AF-like (C-terminal part of AF)	EFWSLAAPQRF-NH_2_		
T-kinin	ISRPPGFSPFR		
**Mix 6**		**Mix 12**	
Substance P	RPKPQQFFGLM-NH_2_	Tail peptide (potential)	ASYYY
Neuropeptide FF precursor	NPAFLFQPQRF-NH_2_	β-casomorphin-7	YPFPGP
γ-Melanocyte-stimulating hormone-like	YVMGHFRWDRF-NH_2_		
VGF(496–507)	PPPRAAPAPTHV	**Mix 13**	
Somatostatin-28(1–12)	SANSNPAMAPRE	Met-enkephalin-RF	YGGFMRF
Cholecystokinin-12	ISDRDYMGWMDF-NH_2_	Urotensin-2B	ACFWKYCV
Neurotensin-like	LYENKPRRPYIL	Melanin-concentrating hormone	DFDMLRCMLGRVYRPCWQV
RFamide-related peptide-1	VPHSAANLPLRF-NH_2_		
Obestatin-13(C-terminal fragment)	LSGAQYQQHGRAL-NH_2_		
Neuropeptide-EI	EIGDEENSAKFPI-NH_2_		

Sequences of peptides are given in the one-letter code. ACTH, Adrenocorticotropic hormone; CART, Cocaine and amphetamine regulated transcript; pQ, pyro-glutamic acid; (M-O), methionine-sulfoxide; (D-K), D-lysine; (K-Ac), acetylated lysine. Sulfo- or phosphotyrosin modifications were not incorporated into peptides (peptides affected: Cholecystokinin-8, Kisspeptin-10, Cholecystokinin-12 and Gastrin). Incidentally, gastrin was not amidated at the C-terminus. Disulfide bridges were introduced in case of the following peptides: Oxytocin, Arg-Vasopressin, Urotensin-2 and Somatostatin-14.

### Modulatory effects of neuropeptides on ASIC1a

**Figure 1:**
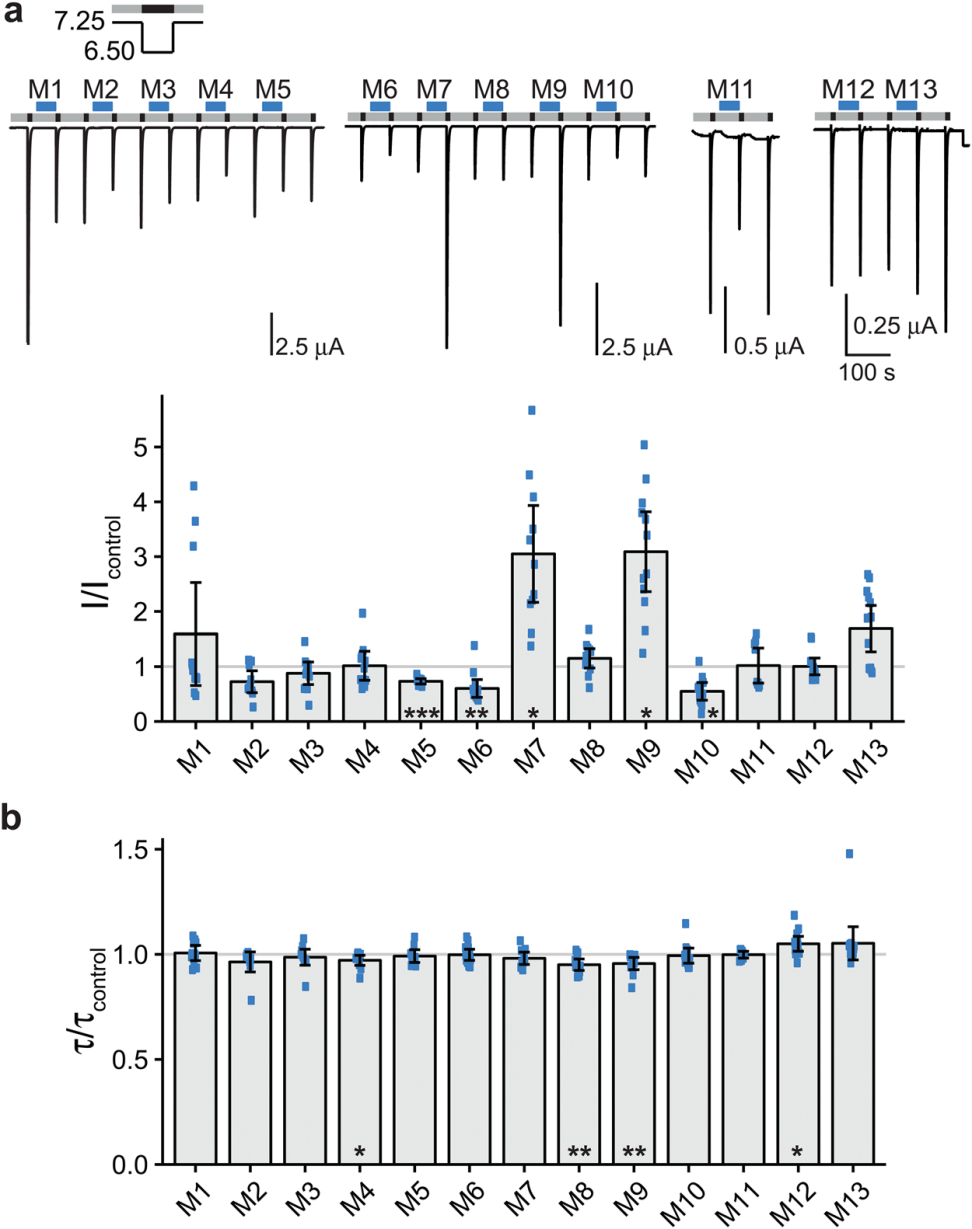
Effects of peptide mixes on ASIC1a current amplitude and desensitization. (**a**) Top, representative current traces of ASIC1a expressing oocytes conditioned with pH 7.25 (grey bars) and activated with pH 6.5 (black bars). Peptide mixes 1–13 (M1-M13) were present in the conditioning solution as indicated by the blue bars with a concentration of 20 μM per individual peptide. Bottom, scatter plot showing ratios of the peak currents after pre-application of peptides (I) to the mean of the two flanking control peak currents (I_control_). Bars show the mean and error bars the SD. (**b**) Scatter plot showing ratios of time constants of desensitization after pre-application of peptides (τ) to the time constants of desensitization of the two flanking control activations (τ_control_). *p < 0.05, **p < 0.01, ***p < 0.001 (paired Student’s t-test).

For ASIC1a, we used a preconditioning pH of 7.25 and an activation pH of 6.5. Under these conditions, current amplitude was somewhat variable also without application of peptides (Fig. 1a). While pre-application of peptide mixes 5, 6 or 10 slightly decreased the ASIC1a current amplitude (mix 5: I/I_control_ = 0.73 ± 0.06, mean ± SD, n = 10, p < 0.001; mix 6: I/I_control_ = 0.60 ± 0.26, n = 13, p < 0.01; mix 10: I/I_control_ = 0.55 ± 0.24, n = 12, p < 0.05; Fig. 1a), pre-application of mixes 7 and 9 increased current amplitude more than 3-fold (mix 7: I/I_control_ = 3.05 ± 1.25, n = 11, p < 0.05; mix 9: I/I_control_ = 3.09 ± 1.10, n = 12, p < 0.05; Fig. 1a). To confirm the decrease in current amplitude by mixes 5, 6 and 10, we split each mix in two submixes, the first submix consisting of the first five peptides and the second of the last five peptides of the original mix. Submixes 5.1, 5.2, 6.1, 6.2, 10.1 and 10.2, however, did not significantly decrease the current amplitude of ASIC1a; mixes 5.2, 6.2, 10.1 and 10.2 even slightly increased current amplitude (Supplementary Fig. 1). We conclude that the change in current amplitude was not very robust and perhaps unspecific and did not investigate further the effect of mixes 5, 6 and 10 on ASIC1a current amplitude.

### Pre-application of dynorphin A(1–13) and dynorphin A(1–17) increased ASIC1a currents

**Figure 2:**
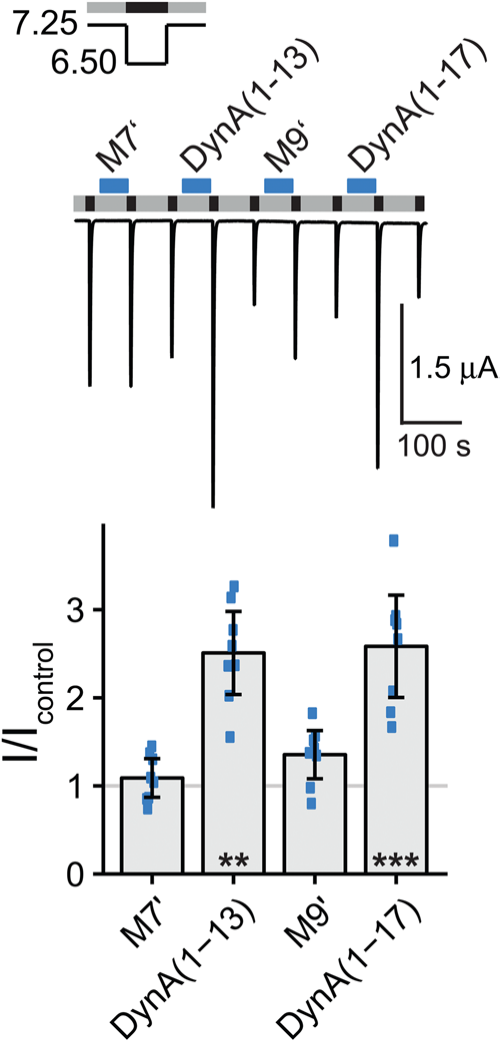
Dynorphin A(1–13) and dynorphin A(1–17) increase ASIC1a current amplitude. Top, representative current trace of an ASIC1a expressing oocyte conditioned with pH 7.25 (grey bars) and activated with pH 6.5 (black bars). Peptide mixes 7′ and 9′ (M7′ and M9′), dynorphin A(1–13) (DynA(1–13)) or dynorphin A(1–17) (DynA(1–17)) were present in the conditioning solution as indicated by the blue bars with a concentration of 20 μM per individual peptide. Mix 7′ contained all peptides of mix 7 except for dynorphin A(1–13), mix 9′ contained all peptides of mix 9 except for dynorphin A(1–17) (See Table 1). Bottom, scatter plot showing ratios of I to I_control_. Bars show the mean and error bars the SD. **p < 0.01, ***p < 0.001 (paired Student’s t-test).

Mixes 7 and 9 contained fragments of dynorphin A (Table 1) which is known to shift the pH dependence of the steady-state desensitization of ASIC1a to more acidic values^16^. Such a shift is expected to increase current amplitude by retaining more channels in the closed state (and thus available for activation) at conditioning pH 7.25. We tested if the presence of dynorphin A fragments could explain the increased current amplitude after pre-application of mix 7 or 9 by removing dynorphin A(1–13) and dynorphin A(1–17) from these mixes. We then individually applied dynorphin A(1–13) (mix 7) and dynorphin A(1–17) (mix 9) and mixes 7 and 9 without dynorphins (mix 7′ and mix 9′) (Fig. 2). As expected, dynorphin A(1–13) and dynorphin A(1–17) robustly increased current amplitude of ASIC1a (dynorphin A(1–13): I/I_control_ = 2.51 ± 0.53, p < 0.01; dynorphin A(1–17): I/I_control_ = 2.58 ± 0.65, p < 0.001; n = 8) while mixes 7′ and 9′ did not (mix 7′: I/I_control_ = 1.09 ± 0.25; mix 9′: I/I_control_ = 1.35 ± 0.31; n = 8; p > 0.05; Fig. 2). We conclude that dynorphin A was responsible for the increased current amplitude after pre-application of mixes 7 and 9. In summary, except for fragments of dynorphin A, we did not identify another peptide that affected ASIC1a current amplitude when pre-applied. We estimated the power of our screen for different effect sizes (see Methods): it had a high power (0.97) to reveal a change of 50% in current amplitude, but a relatively low power (0.49) to detect a small change of 25% (Table 2). Thus, we might have missed peptides that slightly changed current amplitudes.

**Table 2:** Calculation of the statistical power of our screen.

Channel	Condition	Mean	SD	n	50% reduction			25% reduction		
					I_exp_/τ_exp_	d	Power	I_exp_/τ_exp_	d	Power
ASIC1a	Amplitude (μA)	−1.02	0.32	8	−0.51	−1.59	**0.97**	−0.25	−0.79	**0.49**
	τ_des_ (msec)	1376	85	8	688	8.09	**>0.99**	344	4.05	**>0.99**
ASIC1b	Amplitude (μA)	−0.42	0.05	8	−0.21	−4.58	**>0.99**	−0.11	−2.29	**>0.99**
	τ_des_ (msec)	732	55	8	366	6.65	**>0.99**	183	3.32	**0.99**
ASIC3	Amplitude (μA)	−0.24	0.12	8	−0.12	−0.99	**0.67**	−0.06	−0.50	**0.23**
	τ_des_ (msec)	436	87	8	218	2.50	**0.86**	109	1.25	**0.17**

Power was calculated for a 50% reduction of the current amplitude and of τ_des_, and for a 25% reduction, respectively. Power for a 50% or a 25% increase would be identical. See Methods for details.

We analyzed effects on desensitization by determining time constants of desensitization (τ_des_). Four mixes (mix 4, 8, 9 and 12) changed τ_des_ with a p-value p < 0.05, but produced small effect sizes (mix 4: τ/τ_control_ = 0.97 ± 0.03, n = 11, p < 0.05; mix 8: τ/τ_control_ = 0.95 ± 0.04, n = 12, p < 0.01; mix 9: τ/τ_control_ = 0.96 ± 0.04, n = 12, p < 0.01; mix 12: τ/τ_control_ = 1.05 ± 0.06, n = 13, p < 0.05; Fig. 1b). Therefore, we decided to use a stricter significance level (α = 0.001) to decide if a mix should be considered as a relevant modulator of current desensitization of ASICs. For ASIC1a, none of the submixes reached this level of significance; the estimated power of our screen to detect changes in τ_des_ was high (>0.99; Table 2).

### Modulatory effects of neuropeptides on ASIC1b

**Figure 3:**
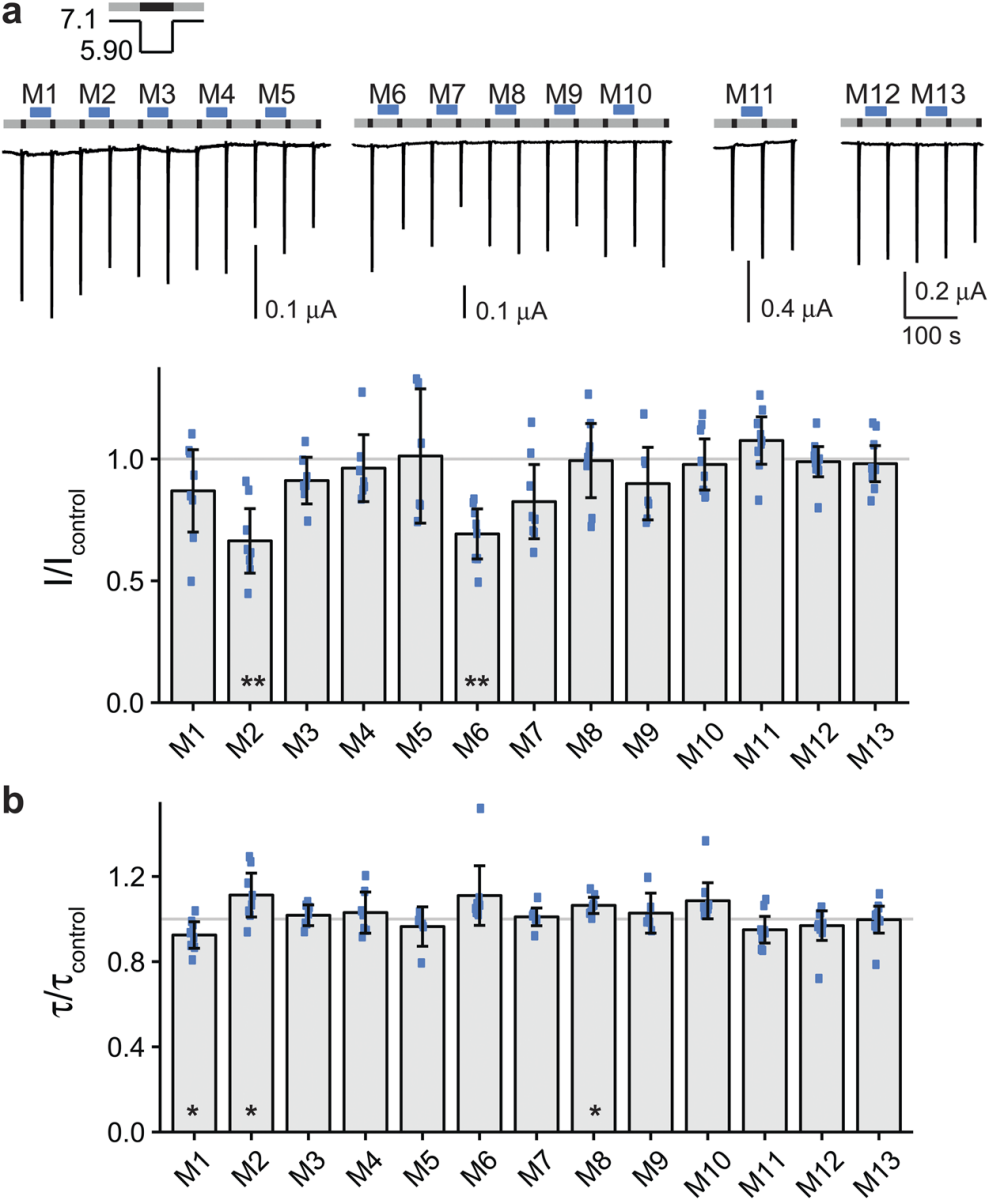
Effect of peptide mixes on ASIC1b current amplitude and desensitization. (**a**) Top, representative current traces of ASIC1b expressing oocytes conditioned with pH 7.1 (grey bars) and activated with pH 5.9 (black bars). Peptide mixes 1–13 (M1-M13) were present in the conditioning solution as indicated by the blue bars with a concentration of 20 μM per individual peptide. Bottom, scatter plot showing ratios of I to I_control_. Bars show the mean and error bars the SD. (**b**) Scatter plot showing ratios of τ to τ_control_. *p < 0.05, **p < 0.01 (paired Student’s t-test).

For ASIC1b, we used pH 7.1 for preconditioning and pH 5.9 for activation. Pre-application of mix 2 and of mix 6 significantly decreased current amplitudes of ASIC1b (mix 2: I/I_control_ = 0.66 ± 0.15; mix 6: I/I_control_ = 0.69 ± 0.12; n = 8; p < 0.01; Fig. 3a). Therefore, we split each of these mixes in half (submixes 2.1, 2.2, 6.1 and 6.2). Only mix 2.1 decreased the current amplitude significantly (I/I_control_ = 0.82 ± 0.04; n = 10; p < 0.05) whereas mixes 2.2, 6.1 and 6.2 did not (Supplementary Fig. 2).

### Pre-application of YFMRFamide reduced current amplitude and slowed desensitization of ASIC1b

**Figure 4:**
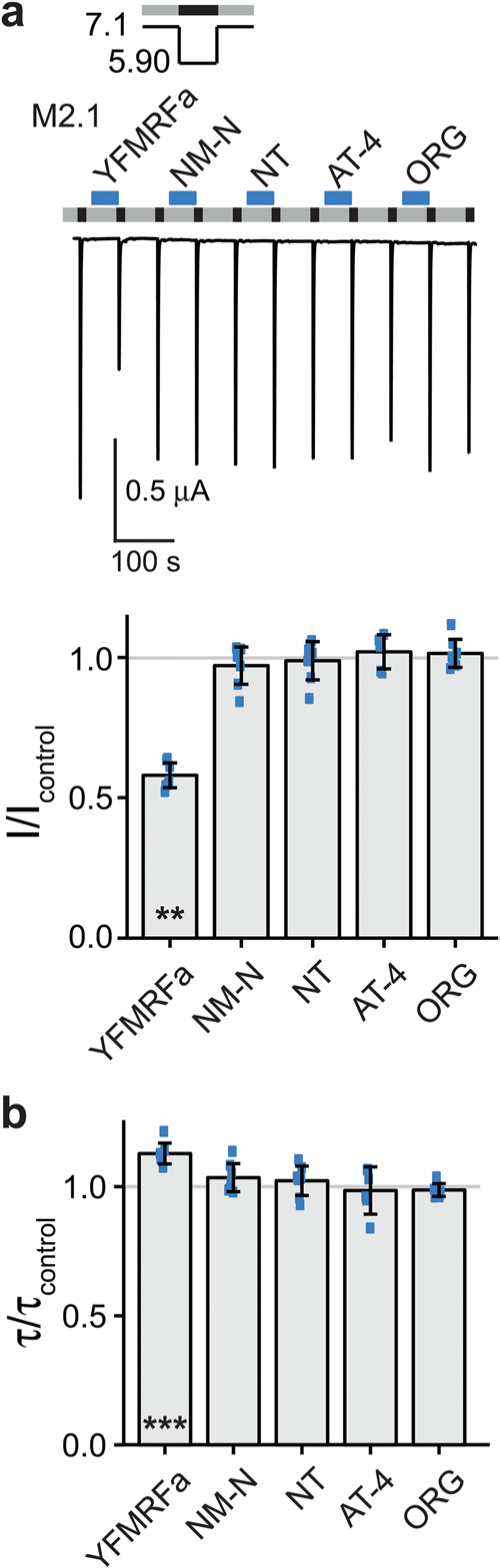
YFMRFamide decreases peak currents and slows desensitization of ASIC1b. (**a**) Top, representative current trace of an ASIC1b expressing oocyte conditioned with pH 7.1 (grey bars) and activated with pH 5.9 (black bars). Individual peptides of mix 2.1 (M2.1) were applied as indicated by the blue bars with a concentration of 20 μM each: YFMRFamide (YFMRFa), neuromedin N (NM-N), NT-2 (fragment analogue of neurotensin) (NT), angiotensin IV (AT-4) and ORG2766 (ORG). Bottom, scatter plot showing ratios of I to I_control_. Bars show the mean and error bars the SD. (**b**) Scatter plot showing ratios of τ to τ_control_. **p < 0.01, ***p < 0.001 (paired Student’s t-test).

Next, we individually pre-applied the five neuropeptides of mix 2.1 (YFMRFamide, neuromedin N, NT-2 (fragment analogue of neurotensin), angiotensin IV and ORG2766). Only pre-application of YFMRFamide significantly decreased the current amplitude (I/I_control_ = 0.58 ± 0.04; n = 7; p < 0.01; Fig. 4a); additionally, it slightly slowed the desensitization of ASIC1b (τ/τ_control_ = 1.13 ± 0.04; n = 7; p < 0.001; Fig. 4b). In contrast, none of the peptide mixes changed τ_des_ of ASIC1b with a high level of significance (p < 0.001) (Fig. 3b). The estimated power of our screen to detect changes in current amplitude and in τ_des_ was high (0.99 or higher; Table 2). We conclude that among the 109 neuropeptides only YFMRFamide modulated ASIC1b. YFMRFamide is related to FMRFamide, which does not strongly modulate ASIC1b^22^. But YFMRFamide is not a mammalian neuropeptide^23^ and therefore no endogenous modulator of ASIC1b.

### Modulatory effects of neuropeptides on ASIC3

**Figure 5:**
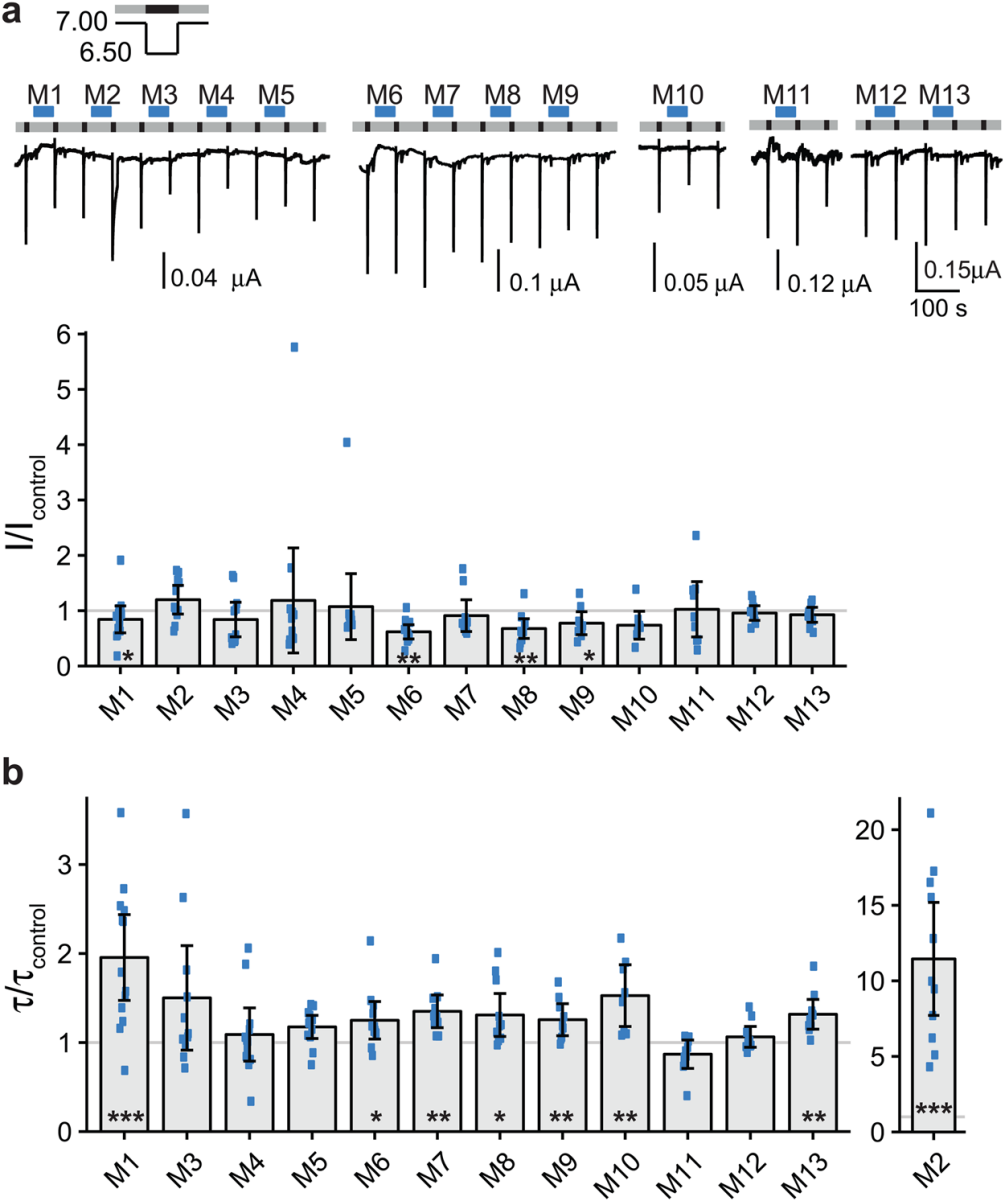
Effect of peptide mixes on ASIC3 current amplitude and desensitization. (**a**) Top, representative current traces of ASIC3 expressing oocytes conditioned with pH 7.0 (grey bars) and activated with pH 6.5 (black bars). Peptide mixes 1–13 (M1-M13) were present in the conditioning solution as indicated by the blue bars with a concentration of 20 μM per individual peptide. Bottom, scatter plot showing ratios of I to I_control_. Bars show the mean and error bars the SD. (**b**) Scatter plot showing ratios of τ to τ _control_. *p < 0.05, **p < 0.01, ***p < 0.001 (paired Student’s t-test).

For ASIC3, we used a conditioning pH of 7.0 and an activation pH of 6.5. Pre-application of mixes 1, 6, 8 and 9 slightly but significantly decreased peak current amplitudes (mix 1: I/I_control_ = 0.84 ± 0.39, n = 13, p < 0.05; mix 6: I/I_control_ = 0.62 ± 0.19, n = 12, p < 0.01; mix 8: I/I_control_ = 0.68 ± 0.25, n = 11, p < 0.01; mix 9: I/I_control_ = 0.77 ± 0.25, n = 9, p < 0.05; Fig. 5a); mix 1 also slowed desensitization kinetics highly significantly (τ/τ_control_ = 1.96 ± 0.77; n = 13; p < 0.001; Fig. 5b). To confirm these effects and to identify individual neuropeptides, we split each mix in half (submixes 1.1, 1.2, 6.1, 6.2, 8.1, 8.2, 9.1 and 9.2). Only mix 1.2 decreased current amplitude significantly (I/I_control_ = 0.80 ± 0.09; n = 8; p < 0.05; Supplementary Fig. 3a). In addition, mix 1.2 also slowed desensitization significantly (τ/τ_control_ = 1.21 ± 0.08; n = 8; p < 0.001; Supplementary Fig. 3b).

### Pre-application of endomorphin-1 and YFMRFa slowed desensitization of ASIC3

**Figure 6:**
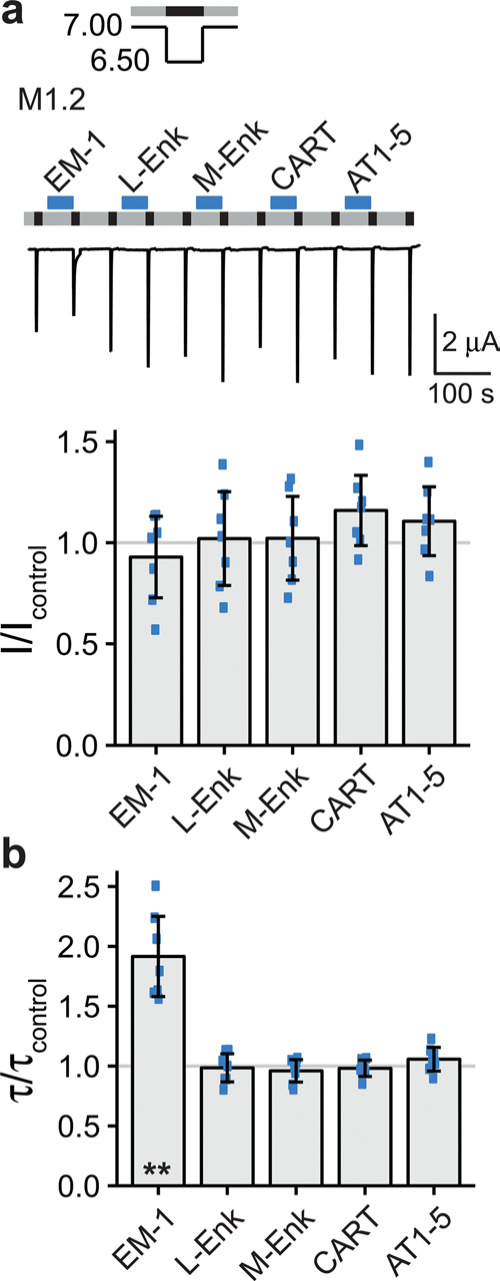
Endomorphin-1 slows the desensitization of ASIC3. (**a**) Top, representative current trace of an ASIC3 expressing oocyte conditioned with pH 7.0 (grey bars) and activated with pH 6.5 (black bars). Individual peptides of mix 1.2 (M1.2) were applied as indicated by the blue bars with a concentration of 20 μM each: endomorphin-1 (EM-1), leu-enkephalin (L-Enk), met-enkephalin (M-Enk), long proCART(55–59) (CART) and angiotensin I/II(1–5) (AT1–5). Bottom, scatter plot showing ratios of I to I_control_. Bars show the mean and error bars the SD. (**b**) Scatter plot showing ratios of τ to τ_control_. **p < 0.01 (paired Student’s t-test).

We next asked which individual neuropeptide decreased current amplitude and slowed desensitization and pre-applied the individual peptides of mix 1.2 (endomorphin-1, leu-enkephalin, met-enkephalin, long proCART(55–59) and angiotensin I/II(1–5)) and identified endomorphin-1 to slow desensitization of ASIC3 (τ/τ_control_ = 1.92 ± 0.34; n = 7; p = 0.001; Fig. 6b). In contrast, none of the individual peptides significantly decreased current amplitude (Fig. 6a). A slowing of ASIC3 desensitization by endomorphin-1 is in line with a recently published article^15^ and can fully account for the slowed desensitization by mix 1 (Fig. 5b).

**Figure 7:**
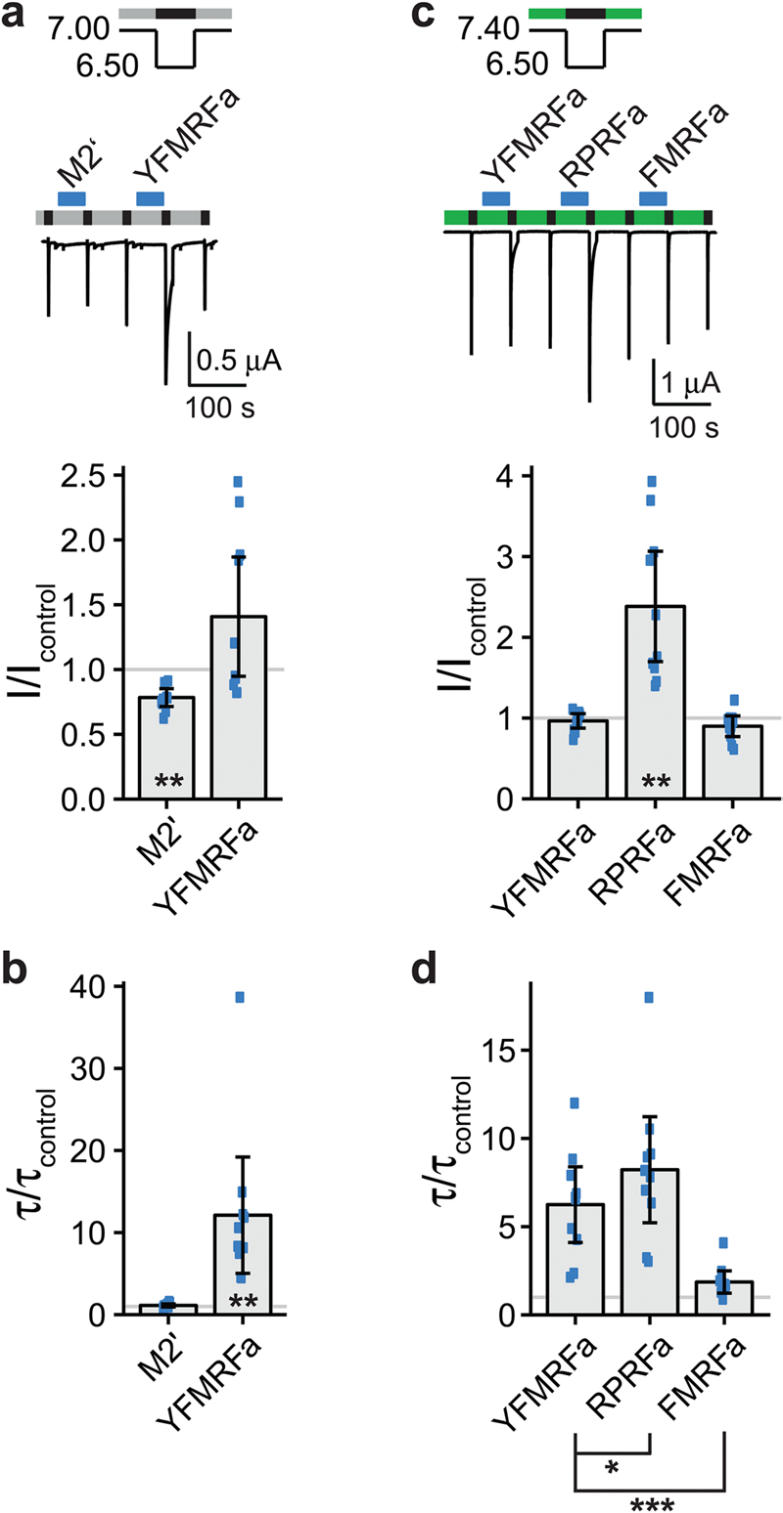
YFMRFamide slows the desensitization of ASIC3. (**a**) Top, representative current trace of an ASIC3 expressing oocyte conditioned with pH 7.0 (grey bars) and activated with pH 6.5 (black bars). Peptide mix 2′ (M2′) and YFMRFamide (YFMRFa) were present in the conditioning solution as indicated by the blue bars with a concentration of 20 μM per individual peptide. Mix 2′ contained all peptides of mix 2 except for YFMRFa. (See Table 1). Bottom, scatter plot showing ratios of I to I_control_. Bars show the mean and error bars the SD. (**b**) Scatter plot showing ratios of τ to τ_control_. (**c**) Top, representative current trace of an ASIC3 expressing oocyte conditioned with pH 7.4 (green bars) and activated with pH 6.5 (black bars). YFMRFamide (YFMRFa), RPRFamide (RPRFa) and FMRFamide (FMRFa) were present in the conditioning solution as indicated by the blue bars with a concentration of 20 μM per individual peptide. Bottom, scatter plot showing ratios of I to I_control_. Bars show the mean and error bars the SD. **p < 0.01 (paired Student’s t-test). (**d**) Scatter plot showing ratios of τ to τ_control_. *p < 0.05, **p < 0.01, ***p < 0.001 (paired Student’s t-test followed by Bonferroni correction).

Like mix 1, mix 2 also significantly slowed desensitization of ASIC3 (mix 2: τ/τ_control_ = 11.45 ± 5.31, n = 11; p < 0.001; Fig. 5b). Mix 2 contained the RFamide YFMRFa, which is a variant of FMRFamide (FMRFa), a peptide known to slow desensitization of ASIC3^12^. This suggested that YFMRFa might be the active compound in mix 2. To confirm this hypothesis, we applied YFMRFa individually and compared its effect to the remaining nine peptides of mix 2 (mix 2′; Fig. 7a). YFMRFa did indeed significantly slow the desensitization of ASIC3 (τ/τ_control_ = 12.12 ± 9.40; n = 10; p < 0.01; Fig. 7b) whereas, surprisingly, the nine remaining peptides decreased the current amplitude significantly (I/I_control_ = 0.78 ± 0.09; n = 10; p < 0.01; Fig. 7a); YFMRFa had no effect on the current amplitude. To find a potentially active peptide within the nine peptides of mix 2′, whose effect on current amplitude might have been masked by YFMRFa, we split mix 2′ in two submixes (mix 2.1′ contained the first four and mix 2.2′ the remaining five peptides; Supplementary Fig. 4). No significant effect on current amplitude could, however, be observed for the two submixes 2.1′ and 2.2′ (Supplementary Fig. 4). We conclude that mix 2 did not contain a peptide, in addition to YFMRFa, that robustly modulated ASIC3. In summary, among the 109 neuropeptides, our screen identified endomorphin-1 and YFMRFa as modulators of ASIC3. The estimated power of our screen to detect changes in current amplitude was relatively low (0.67 for a change of 50%) and to detect changes in τ_des_ it was modest (0.86 for a 50% change; Table 2). Thus, our screen could only identify peptides with a strong effect on current amplitude or on τ_des_ of ASIC3 (change of at least 50%).

Since modulation of ASIC3 by YFMRFa has not been previously reported, we compared its effect on ASIC3 to two other RFamides that are known to slow desensitization of ASIC3: the conorfamide RPRFamide (RPRFa) and FMRFa^13^. To evaluate modulation, we used preconditioning pH 7.4 and activation pH 6.5; RFamides were pre-applied individually. RPRFa significantly increased the peak current (I/I_control_ = 2.38 ± 0.91; n = 10; p < 0.01), whereas YFMRFa and FMRFa did not (Fig. 7c). Concerning desensitization, RPRFa had a stronger effect on ASIC3 than YFMRFa (RPRFa: τ/τ_control_ = 8.23 ± 3.99; YFMRFa: τ/τ_control_ = 6.25 ± 2.85; n = 10; p = 0.04; Fig. 7d) but YFMRFa had a stronger effect than FMRFa (FMRFa: τ/τ_control_ = 1.86 ± 0.84; n = 10; p < 0.001; Fig. 7d). Thus, the three peptides slowed desensitization with the following rank order RPRFa > YFMRFa > FMRFa.

### Direct effects of neuropeptides on ASIC4 and BASIC

**Figure 8:**
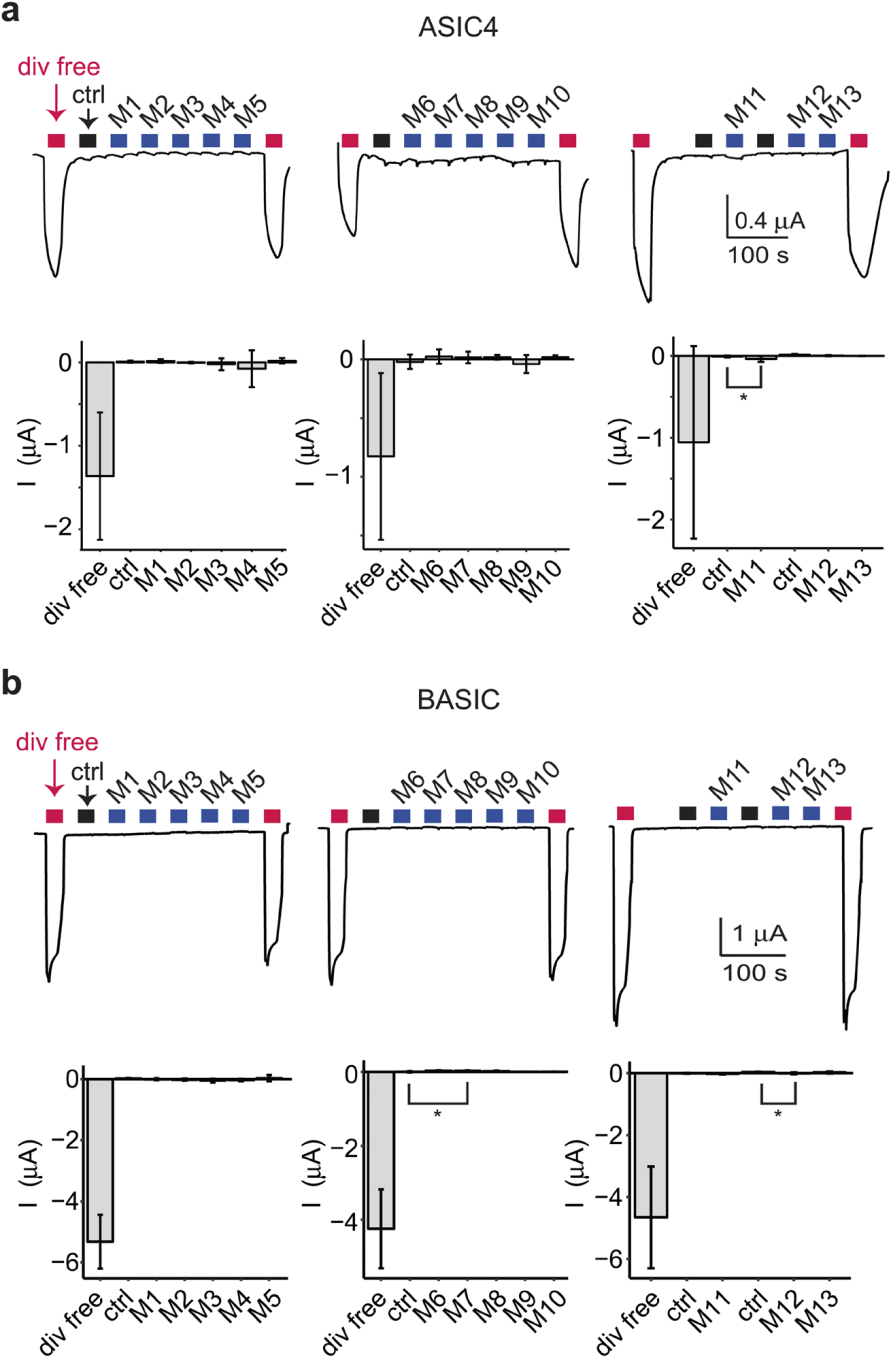
There is no direct agonist for ASIC4 and BASIC among the 109 neuropeptides. (**a,b**) Top, representative current traces of ASIC4 (a) or BASIC (b) expressing oocytes activated with divalent free solution (div free; red bars). Controls (ctrl; black bars) before each peptide mix (M; blue bars) contained the solvent in its respective concentration. Peptides were applied at pH 7.4. Bottom, bar graphs showing the mean current amplitudes; error bars show the SD. *p < 0.05 (paired Student’s t-test followed by Bonferroni correction).

We then screened the 109 neuropeptides on ASIC4- or BASIC-expressing oocytes to identify a direct agonist of these two channels. The peptides were applied at a pH of 7.4 in the same 13 mixes as before (Table 1). Divalent free bath solution opens ASIC4 and BASIC^24,25^. Therefore, we checked for channel expression with control activations with divalent free solution at the beginning and at the end of experiments. Solution containing the respective vehicle (water, NH_4_Cl or DMSO) was applied as a control for solution exchange artifacts. Mix 11 induced significant currents of ASIC4-expressing oocytes relative to control solution (p < 0.05; Fig. 8a). The current amplitude was tiny, however, for a high concentration (20 μM) of individual peptides (I_mix11_ = −0.04 ± 0.03 μA; I_control_ = −0.01 ± 0.01 μA; n = 6; p < 0.05; Fig. 8a). Therefore, we did not consider this effect size as physiologically relevant and did not investigate mix 11 on ASIC4 further. Mix 7 and mix 12 induced small but significant currents on BASIC expressing oocytes (mix 7: I_mix7_ = 0.03 ± 0.01 μA; I_control_ = −0.01 ± 0.02 μA, n = 6, p < 0.05; mix 12: I_mix12_ = -0.01 ± 0.02 μA; I_control_ = −0.03 ± 0.01 μA; n = 6; p < 0.05; Fig. 8b). The current that was induced by mix 7 however was an outward current and the current induced by mix 12 was quite small. Therefore, we decided not to further investigate the effect of mix 7 and mix 12 on BASIC. We conclude that the 109 neuropeptides do not contain a direct agonist of ASIC4 or BASIC.

## Discussion

Our study was motivated by the question whether short neuropeptides act as direct agonists of ASICs, with an emphasis on the orphan receptors ASIC4 and BASIC. Since the peptides that activate established peptide-gated DEG/ENaCs contain four (FMRFamide, the ligand of FaNaC)^17^, seven (Hydra-RFamides, the ligands of HyNaCs)^18^ or nine to ten amino acids (myoinhibitory peptides, the ligands of MGIC)^20^, and to limit the financial costs of our screen, we restricted it to peptides that contain less than 20 amino acids. In a recent screen of 121 neuropeptides from the marine annelid *Platynereis*, we identified myoinhibitory peptides as agonists of MGIC^20^, illustrating the sensitivity of our screening system. In our new screen, we used rat ASICs and chose rat neuropeptides from NeuroPep, a comprehensive database that at its recent release was the most complete neuropeptide database^21^. Thus, although we cannot exclude that some small peptides were missing from our screen, it is likely that most known small neuropeptides were included. Therefore, we conclude that short neuropeptides (<20 amino acids) are unlikely direct agonists of ASICs. We cannot exclude, however, that a short peptide currently not contained in NeuroPep or a longer peptide (>20 amino acids) directly activates an ASIC.

In addition, we used our panel of peptides to screen for new modulators of ASIC1a, ASIC1b and ASIC3. To be able to screen many peptides on several ASICs, we used an application protocol that allowed us to capture at the same time modulation of activation, of steady-state desensitization and of acute desensitization. With this protocol, however, current amplitudes were somewhat variable also under control conditions, limiting the sensitivity of our screen. To keep the sensitivity of our screen as high as possible we used a significance level of 0.05 and no correction for multiple testing. Instead, we performed follow-up tests of positive mixes to reduce the false positive rate. This is illustrated by cases, in which original peptide mixes appeared to change current amplitudes but in which this change could not be confirmed by peptide submixes. Although, we cannot rule out that some peptides were active only when co-applied with each other and that this effect was lost upon un-mixing, we considered in these cases the initial change of current amplitude as a false positive result. The variability of current amplitude can at least partially be explained by a conditioning pH that desensitized approximately 50% of the channels. Due to the extreme steepness of steady-state desensitization curves of ASICs (Hill coefficient for ASIC1a ~11)^6^, at this pH a minor change in pH in our recording chamber would have immediately affected current amplitude. Similarly, due to the large size of oocytes and the comparatively slow solution exchange, acute desensitization of ASICs cannot be measured with a high precision and reproducibility^26^. Moreover, the size of effects on desensitization was small. This led us to apply a more stringent criterion to assess significance of changes in τ_des_ (p < 0.001) than of changes in current amplitudes (p < 0.05). Finally, since we used mixes of peptides, we cannot exclude that peptides within one mix interfered with each other, potentially masking a modulatory effect. We have recently shown, however, that by screening of peptide mixes, novel peptide ligands for DEG/ENaCs can be identified^20^.

The limited sensitivity of our screen is also illustrated by the fact that the power of our screen to detect a relatively small change in current amplitude of 25% was high only for ASIC1b, but low for ASIC1a and ASIC3 (Table 2). The power to detect a robust change in current amplitude of 50% was high also for ASIC1a, but relatively low for ASIC3. The power to detect changes of τ_des_ was generally high, with the exception of small changes of τ_des_ for ASIC3 (Table 2). This limited sensitivity for ASIC3 can explain why we missed NPFF, a known modulator of ASIC3, which slows desensitization^12,22,27^ and which was contained in mix 3 (NPFF) (Table 1), but which has rather small effects^12,22,27^.

Irrespective of the limitations of our screen (uncomprehensive set of peptides, limited sensitivity, use of peptide mixes), we identified several known modulators of ASICs, dynorphin A^16^ and endomorphin-1^15^, demonstrating that we were able to identify strong modulators of ASICs. Thus, although we might have missed some peptides with a small modulatory effect on ASICs, we conclude that most small neuropeptides robustly modulating ASICs are already known.

In summary, reporting mainly negative results, we believe that our screen will help to focus future research on the interaction of ASICs with known modulatory neuropeptides and advances our understanding of the interaction of ASICs with neuropeptides.

## Methods

### Peptides

Peptide sequences were retrieved from the online database NeuroPep on July 25, 2016. In some cases, peptide sequences were adjusted relative to the sequences supplied by NeuroPep (see Table 1): the two peptides hexarelin and cortistatin-14 were not included in our screen and sequences of ORG2766 and ACTH4–10 were corrected according to the literature. Although indicated as “rat” peptides in the database, some of the peptides have an uncertain origin. YFMRFamide, for example, is not a mammalian neuropeptide.

Neuropeptides were purchased from GenScript (New Jersey, United States) as either lyophilized powders or crystals with a purity of >95%. RPRFamide and FMRFamide were obtained from ProteoGenix (Schiltigheim, France) and kept in aqueous stock solutions. We dissolved the neuropeptides either in ultrapure degased water (mixes 1–10), in water containing 0.75–3% (w/v) NH_3_ (mix 11) or in dimethyl sulfoxide (DMSO) (mixes 12 and 13) to obtain a concentration of 2 to 10 mM per peptide. Up to ten peptides were then pooled to form stock solutions of the 13 mixes. The composition of each mix is indicated in Table 1. The concentration of individual peptides in the stock solution was 200 μM for mixes 1–10, 1 mM for mix 11, 5 mM for mix 12 and 3.33 mM for mix 13. Stock solutions of individual peptides or of peptide mixes were kept at −80 °C.

All other chemicals were purchased from Sigma-Aldrich (Munich, Germany) or Carl Roth GmbH (Karlsruhe, Germany).

### Molecular biology and handling of oocytes

Animal care and experimental protocols for female *Xenopus laevis* frogs were approved by the Landesamt für Natur, Umwelt und Verbraucherschutz of North Rhine-Westphalia (LANUV NRW) and were performed in accordance with LANUV NRW guidelines. cRNA synthesis and preparation of oocytes was described previously^20^. Stage V and VI oocytes were injected with the following amounts of cRNA: 0.02 ng of rat ASIC1a, 0.41 ng of rat ASIC1b, 3.3 ng of rat ASIC3, 1.7–6.7 ng of rat ASIC4 or 26 ng of rat BASIC. After injection, oocytes were kept at 19 °C in Oocyte Ringer-2 (OR-2; in mM: 82 NaCl, 2.5 KCl, 1 Na_2_HPO_4_, 1 MgCl_2_, 1 CaCl_2_, 0.5 g/l PVP, 5 HEPES; pH 7.3), except for BASIC-expressing oocytes which were kept in low-Na^+^ OR-2 (in mM: 77.5 NMDG, 5 NaCl, 2.5 KCl, 1 Na_2_HPO_4_, 1 MgCl_2_, 1 CaCl_2_, 0.5 g/l PVP, 5 mM HEPES; pH 7.3).

### Electrophysiological recordings

Electrophysiological recordings on *Xenopus laevis* oocytes were performed 24–72 h after injection with a TurboTec 03X two electrode voltage clamp amplifier (npi electronic, Tamm, Germany). Fast solution exchange was provided by a programmable pipetting robot (screening tool; npi electronic)^28^. Data were filtered at 20 Hz and sampled at 500 Hz. Data acquisition was performed using CellWorks 6.2.1 (npi electronic). The holding potential was −70 mV.

Bath solutions were freshly prepared each day by adding peptide mixes or single peptides to bath solution at a final concentration of 20 μM of each peptide. Bath solution contained the following (in mM): 140 NaCl, 1.8 CaCl_2_, 1 MgCl_2_ and 10 HEPES or MES (pH < 6.6); solutions with a pH of 7.0–7.25 contained 20 HEPES. For mix 11, bath solution contained also NH_3_ (final concentration 0.042%, w/v) and for mixes 12 and 13 DMSO (final concentration 0.4% DMSO in mix 12 and 0.6% DMSO in mix 13; v/v). Peptide-containing solutions and control solutions only differed in the presence or absence of neuropeptides. Peptide-containing solutions were titrated by adding NaOH and HCl immediately before the measurements. We have chosen a pH that desensitizes 50% of the channels based on values published previously by our group^6,13^; those pH values were verified for the specific set-up used in control experiments at the beginning of this study. Different peptide mixes were applied to half of the oocytes expressing ASIC1a, ASIC1b or ASIC3 in numerical order (e.g. from mix 1 to mix 5) and to the other half in inverse order (e.g. from mix 5 to mix 1).

### Data analysis

Data was analyzed using CellWorks Reader 6.2.2 (npi electronic) or Igor Pro 5.0.3 (WaveMetrics, Portland, USA); statistical analysis was performed in Microsoft Excel or R 3.4.1^29^ and plots were generated using the ggplot2 package^30^ in R. In some cases, the baseline was corrected with the software.

For ASIC1a, ASIC1b and ASIC3, p-values were determined by paired Students t-test. A t-test was performed for each peptide application; the comparison was made between peak current amplitudes after pre-application of peptide and the mean of the peak current amplitudes of the two flanking activations after pre-application of vehicle. For reasons of clarity, instead of plotting the absolute values of currents after pre-application of peptide and vehicle we plotted the ratios (I/I_control_). Current decays were fit to a single exponential function in Igor Pro to obtain time constants of desensitization (τ_des_). Statistical analysis and plotting of τ_des_ were performed in analogy to the peak currents. To detect false-positive results, we performed a second round of screening with submixes.

For ASIC4 and BASIC, p-values were also determined by paired Students t-test. In contrast to ASIC1a, ASIC1b and ASIC3, only one control activation with vehicle was performed at the beginning of each measurement. To correct for multiple testing, we applied Bonferroni correction. A t-test was performed for each peptide application; the comparison was made between current amplitudes during peptide application and current amplitudes during application of vehicle. Plots depict absolute current amplitudes.

All results are reported as mean ± standard deviation (SD). Experiments were performed with oocytes of at least two different batches of oocytes.

We estimated the statistical power of our screen for a modulatory effect on ASIC1a, ASIC1b and ASIC3 for a relative current change of 50% and of 25%. The expected current, I_exp_, was thus obtained by multiplying the average control current amplitude by 0.5 or 0.25, respectively. Since the t-test compared current amplitudes after pre-application of peptide with control applications (see above), we determined the standard deviation for control currents without peptide. First, we calculated the standard deviation of control currents for each oocyte. These standard deviations were then averaged for each ASIC. Note that we assumed that the standard deviation was similar for control measurements and measurements with pre-application of peptides. I_exp_ was then divided by the standard deviation of the corresponding control measurements to yield Cohen’s *d* as a measure of the effect size: *d* = I_exp_/SD. The ‘pwr’ package of R was used to calculate the power for specific values of *d*, the average number of recordings *n* (n = 8) and the α-level (α = 0.05) for each channel. The statistical power for relative changes of τ_des_ was calculated in a similar way, except that the α-level was adjusted (α = 0.001). Values for the statistical power are indicated in Table 2.

## Electronic supplementary material

Supplementary Information

## Data Availability

All data are available from the corresponding author upon request. All relevant data are within the paper.
