# Supplementary material for: Screening of 109 neuropeptides on ASICs reveals no direct agonists and dynorphin A, YFMRFamide and endomorphin-1 as modulators
Source: Sci Rep. 2018 Dec 20;8:18000. doi: 10.1038/s41598-018-36125-5 (PMC6301962; doi:10.1038/s41598-018-36125-5)
Supplement: Supplementary file 1 — Supplementary Information [file 41598_2018_36125_MOESM1_ESM.pdf]

Screening of 109 neuropeptides on ASICs reveals no direct agonists and dynorphin A, YFMRFamide and endomorphin-1 as modulators

Anna Vyvers, Axel Schmidt, Dominik Wiemuth, Stefan Gründer

Supplementary information

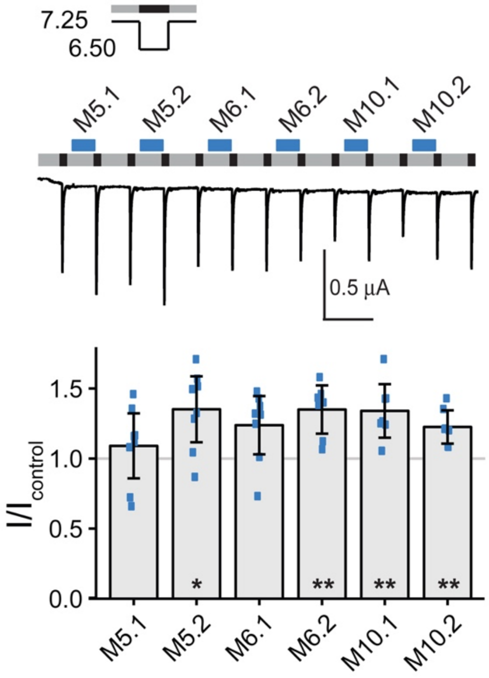

Supplementary Figure 1. Effect of submixes on ASIC1a current amplitude. Top, representative current trace of an ASIC1a expressing oocyte conditioned with pH 7.25 (grey bars) and activated with pH 6.5 (black bars). Peptide mixes (M) 5.1, 5.2, 6.1, 6.2, 10.1 and 10.2 were present in the conditioning solution as indicated by the blue bars with a concentration of 20 µM per individual peptide. Bottom, scatter plot showing ratios of I to $I_{control}$. Bars show the mean and error bars the SD. *$p < 0.05$, **$p < 0.01$ (paired Student's t-test).

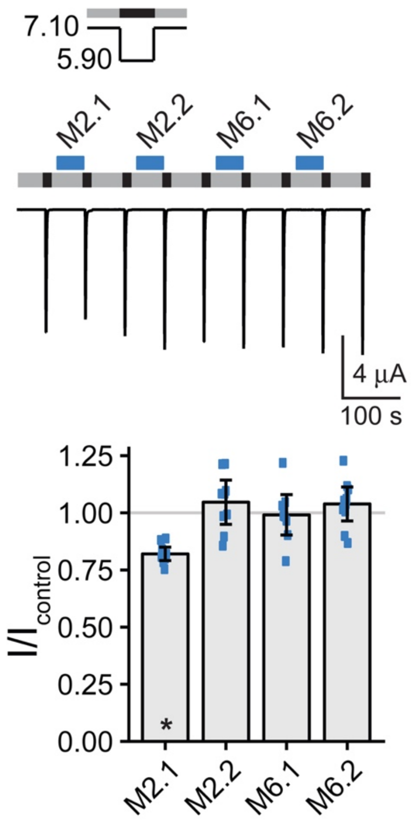

Supplementary Figure 2. Effect of submixes on ASIC1b current amplitude. Top, representative current trace of an ASIC1b expressing oocyte conditioned with pH 7.1 (grey bars) and activated with pH 5.9 (black bars). Peptide mixes (M) 2.1, 2.2, 6.1 and 6.2 were present in the conditioning solution as indicated by the blue bars with a concentration of 20 μM per individual peptide. Bottom, scatter plot showing ratios of I to $I_{control}$. Bars show the mean and error bars the SD. *$p < 0.05$ (paired Student's t-test).

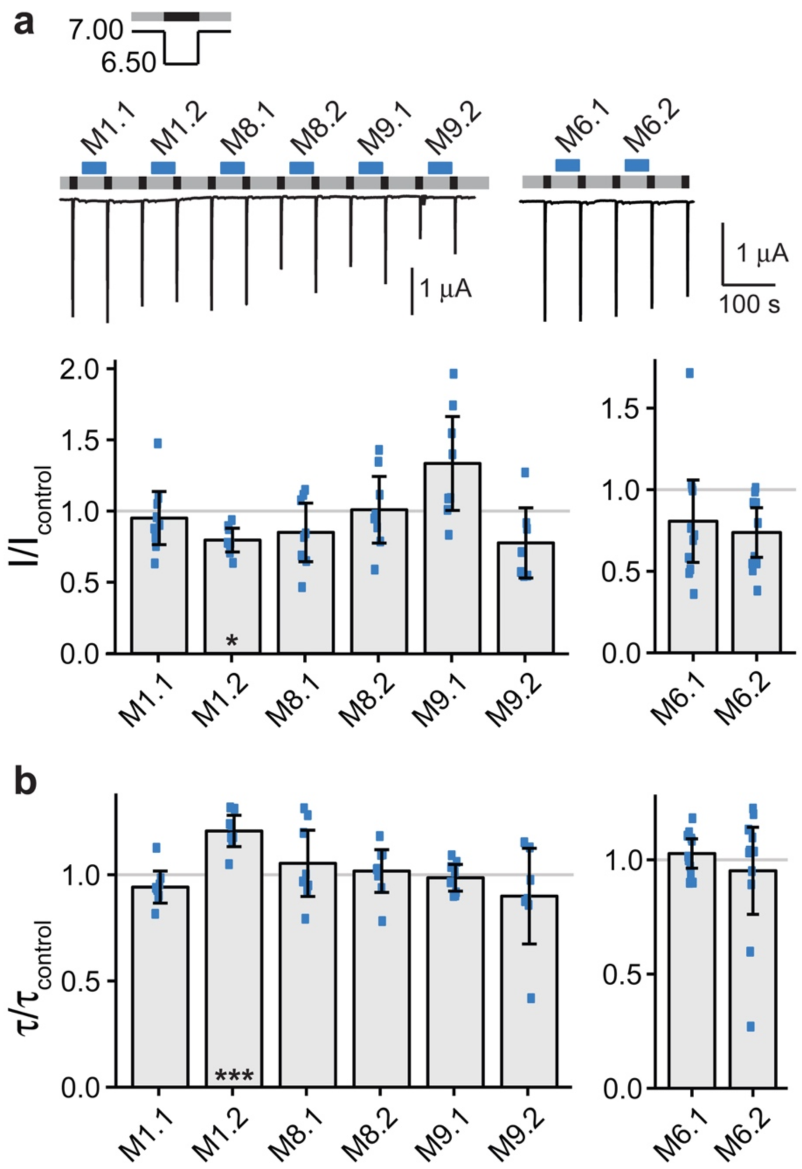

Supplementary Figure 3. Effect of submixes on ASIC3 current amplitude and desensitization. (a) Top, representative current traces of ASIC3 expressing oocytes conditioned with pH 7.0 (grey bars) and activated with pH 6.5 (black bars). Peptide mixes (M) 1.1, 1.2, 8.1, 8.2, 9.1, 9.2, 6.1 or 6.2 were present in the conditioning solution as indicated by the blue bars with a concentration of 20 µM per individual peptide. Bottom, scatter plot showing ratios of I to $I_{control}$. Bars show the mean and error bars the SD. **(b)** Scatter plot showing ratios of τ to $\tau_{control}$. *$p < 0.05$, ***$p < 0.001$ (paired Student's t-test).

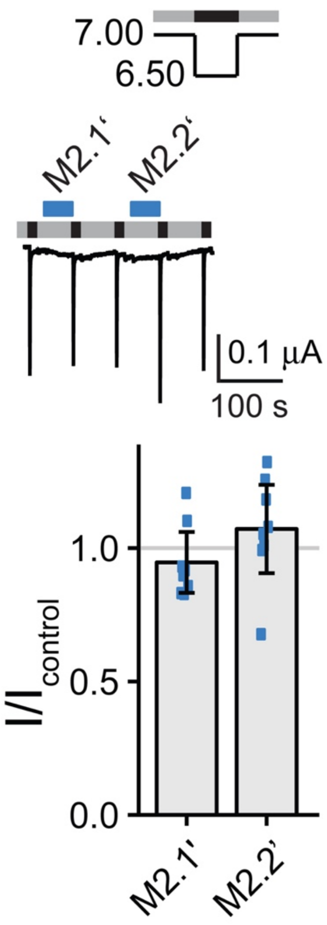

Supplementary Figure 4. Effect of submixes on ASIC3 current amplitude. Top, representative current trace of an ASIC3 expressing oocyte conditioned with pH 7.0 (grey bars) and activated with pH 6.5 (black bars). Peptide mixes 2.1' and 2.2' (M2.1', M2.2') were present in the conditioning solution as indicated by the blue bars with a concentration of 20 µM per individual peptide. Bottom, scatter plot showing ratios of I to $I_{control}$. Bars show the mean and error bars the SD.
